# Effective Removal of Congo Red by Triarrhena Biochar Loading with TiO_2_ Nanoparticles

**DOI:** 10.1155/2018/7670929

**Published:** 2018-06-04

**Authors:** Peng Yu, Tao Hu, Hong Hui Chen, Fangfang Wu, Hui Liu

**Affiliations:** ^1^College of Science, Hunan Agricultural University, Changsha 410128, China; ^2^Changde Xinrui New Material Co. Ltd., Changde 415004, China

## Abstract

A composite of pyrolytic Triarrhena biochar loading with TiO_2_ nanoparticles has been synthesized by the sol-gel method. The composite shows a well-developed hollow mesoporous and macropore structure as characterized by XRD, BET, and SEM. When used as an absorbent to remove Congo red from aqueous solution, it was found that as-prepared composite performed better absorption capacity than single biochar or TiO_2_. The results suggest that biochar loading with TiO_2_ could be promisingly implemented as an environmentally friendly and inexpensive adsorbent for Congo red removal from wastewater.

## 1. Introduction

With the rapid development of global printing and dyeing industry (PDI) for decades, a variety of dyes are used in knitting silk, cotton, and so on [1–3]. Considerable industrial wastewater including dyes would have been generated during the processing of PDI [4–8]. Congo red 1-naphthalenesulfonic acid, 3, 3′-(4, 4′-biphenylenebis (azo) bis (4-amino) disodium salt, as one of the most widely used direct dyes in PDI for its high chromaticity, is the critical source contamination of wastewater with high chemical oxygen demand and high biological toxicity [9–14]. Therefore, it is of great significance to remove Congo red from aqueous solution.

It has been reported that physical adsorption is the dominant method for Congo red removal. The materials with a high-specific surface area and an abundant pore structure are explored to absorb Congo red to decontaminate industrial wastewater [15, 16]. For instance, activated carbon (AC), possessing a large specific surface area [17], good environmental friendliness [18], and mechanical stability [19], is a promising material for wastewater treatment [20]. However, its application is limited by high energy consumption and large greenhouse gas emissions during an AC preparation process [21]. Recently, biochar (BC), a precursor of AC, has attracted many researchers' attention [22]. It has a pore structure with a large surface area and good environmental friendliness as well as AC. In addition, BC could usually be prepared by pyrolysis of biological organic materials under a moderate temperature with low cost. And pyrolytic BC could keep the original structure of the rich organic functional group and could be modified easily [23, 24]. When BC is used as an adsorbent to remove Congo red, its organic functional group would interact weakly with the organic molecule of Congo red. Hence, BC might show better research prospects than AC in terms of waste water purification, heavy metal ion adsorption, and soil restoration [24–28]. However, BC mostly exhibits a micropore and mesoporous structure, which seldom matches Congo red macromolecule. Therefore, it is necessary to enlarge the pore size of BC to enhance its absorption capacity.

In this work, TiO_2_ nanoparticles have been loaded to modify BC. Nanosized TiO_2_ is also an excellent adsorbent, which has been studied in the aspect of pollution absorption [29]. However, its adsorption capacity is weakened due to the nature of its easy aggregation. Here, OH in the surface of TiO_2_ generated via Ti-R4 + 4H2O ➔Ti(OH)4 + 4R(OH) would interact weakly with C=O of BC. It is predicted that the interaction would introduce TiO_2_ to enter the pore structure to enlarge the pore size of BC and avoid aggregation of TiO_2_ [30]. Therefore, BC loading with TiO_2_ nanoparticles (BC@TiO_2_) has been synthesized and used as an absorbent to remove Congo red from aqueous solution. The adsorption performance of BC@TiO_2_ composite is expected to be greatly enhanced due to the synergistic adsorption combination of TiO_2_ and BC.

## 2. Materials and Methods

### 2.1. Material Preparation

BC was obtained by the pyrolysis of Triarrhena stalks. Triarrhena with a short growth cycle is abundant in the south of the Yangtze River. Triarrhena stalks were collected from Hunan Agricultural University (Changsha, China) miscanthus resources nursery, washed in distilled water to remove adhering impurities, and then chopped into small pieces (1~1.5 cm). Finally, Triarrhena stalk pieces were heated to obtain BC in the nitrogen atmosphere at different temperatures of 300°C, 400°C, and 500°C, named as BC-300°C, BC-400°C, and BC-500°C, respectively.

The composite of BC@TiO_2_ was prepared using the following procedure. Firstly, anhydrous ethanol was taken in a beaker, and four n-butyl titanates was dripped dropwise using a burette under magnetic stirring to form yellow clear solution A. Secondly, solution B was prepared by adding acetic acid and distilled water into ethanol in another beaker and then adjust its pH value to 3 by dropping hydrochloric acid. Thirdly, solution A is slowly dripped into solution B under magnetic stirring at a water bath of ambient temperature. Triarrhena BC was added into the mixing solution gel after finishing dropping and continually stirred at a water bath of 80°C for half an hour. And then as-prepared gel was dried at 105°C in an oven for 12 h. Lastly, the dried materials were crushed and burned in a nitrogen atmosphere at different temperatures (300°C, 400°C, and 500°C, resp.) to obtain BC@TiO_2_ composite, named as BC@TiO_2_-300°C, BC@TiO_2_-400°C, and BC@TiO_2_-500°C, respectively. For comparison, TiO_2_-300°C, TiO_2_-400°C, and TiO_2_-500°C were prepared in the same procedure without BC.

### 2.2. Microstructural Characterization

The microstructure of samples was characterized by a Shimadzu X-ray 6000 diffractometer (XRD) with Cu _K*α*_ radiation at 40 kV, 30 mA, and a Supra40 Carl Zeiss scanning electron microscopy (SEM) coupled with EDS, determining the mapping distribution of C, O, and Ti. The BET surface area (*S*_BET_) and micropore width were analyzed by the specific surface and aperture analyzer (Quadrasorb SI series, Quantachrome, USA) using nitrogen adsorption at 77 K.

### 2.3. Adsorption Measurements

As-prepared BC, TiO_2_, and BC@TiO_2_ were used as adsorbents for Congo red adsorption study. 0.015 g sample was added into 100 mL Erlenmeyer flasks containing 20 mL Congo red (analytical purity, SSS Reagent Co. Ltd., Shanghai, China), and then Erlenmeyer flasks were placed on a rotary shaker with 150 rpm at 25°C. For the adsorption kinetic study, the suspensions were immediately filtered through a 0.45 *μ*m filter at each sampling time and subjected to analyze. Congo red absolute adsorbed capacity (*q*_e_) was calculated by (1) based on mass balance, while the adsorption efficiency was calculated by (2). And then the optimum amount of the optimum adsorbent was determined under the same conditions.

(1) $$\begin{array}{c} q_{e}=\frac{\left(C_{0}-C_{e}\right)V}{m}, \end{array}$$

(2) $$\begin{array}{c} p=\frac{\left(C_{0}-C_{e}\right)V}{C_{0}}\times 100\%, \end{array}$$

where *C*_0_ (mg·L^−1^) is referred to the initial Congo red concentration, *C*_e_ (mg·L^−1^) is referred to the Congo red concentration at equilibrium, *V* (L) is the solution volume, and *m* (g) is concerned to the weight of the adsorbent.

All adsorption results were calculated from an average of three independent experimental results, and the maximum deviations from the average (error bars) were also indicated.

## 3. Results and Discussion

**Figure 1:**
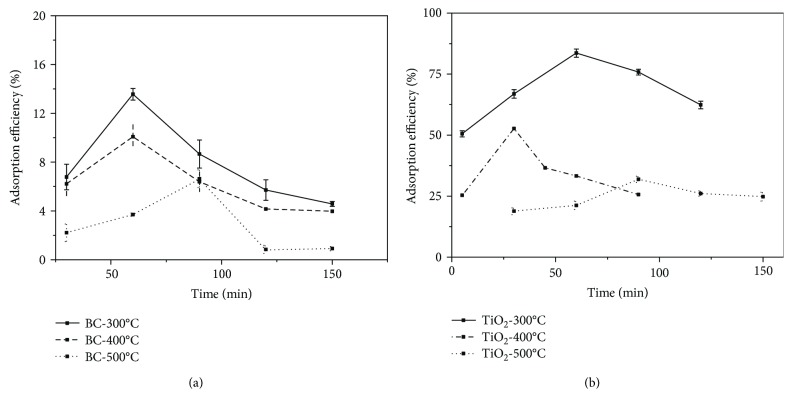
The adsorption efficiency of (a) BC and (b) TiO_2_ synthesized at 300°C, 400°C, and 500°C.

To determine the optimum temperature of heating treatment, Figure 1 shows the adsorption efficiency of as-prepared BC and TiO_2_ prepared at different temperatures of 300°C, 400°C, and 500°C. It could be revealed that a rapid initial sorption could be observed during the first 90 min and the adsorption capacity is basically saturated for all BC and TiO_2_ samples. In more detail, BC-300°C performs the largest relative adsorption capacity of 13.56% after about 60 min, while BC-400°C of 10.08% after 60 min and BC-500°C after 90 min as shown in Figure 1(a). TiO_2_ performs the same trend that TiO_2_-300°C has the largest relative adsorption efficiency of 83.59%, followed by 52.64% of TiO_2_-400°C and 31.89% of TiO_2_-500°C. Therefore, it is determined that our target material could be prepared at the optimum temperature of 300°C with low energy consumption and would have excellent adsorption performance.

**Figure 2:**
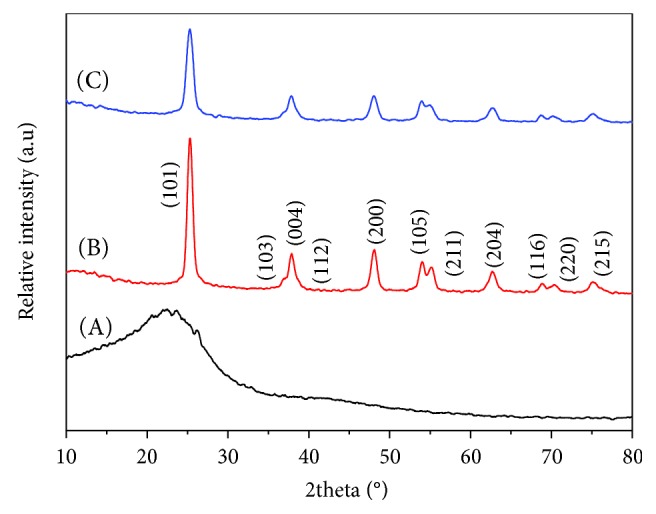
XRD patterns of A BC-300°C, B TiO_2_-300°C, and C BC@TiO_2_-300°C.

Figure 2 shows the XRD patterns of BC-300°C, TiO_2_-300°C, and BC@TiO_2_-300°C. There is a broad diffraction peak (2*θ* = 20°~27°) which is attributed to disordered structures of BC as shown in Figure 2 A. In Figures 2 B and 2 C, the diffraction peaks at 2θ of 25.36°, 37.91°, 48.16°, 54.05°, 55.20°, 62.86°, 68.97°, 70.48°, and 75.28° belong to (101), (004), (200), (105), (211), (204), (116), (220), and (215) of anatase TiO_2_, respectively (number 01-084-1286). The broad diffraction peak of C could not be observed because of an overlapping high crystalline TiO_2_ peak of (101) in BC@TiO_2_-300°C in Figure 2 C. Compared to Figures 2 B and 2 C, the diffraction peaks of TiO_2_ widen and weaken significantly. Based on peak profile analysis using a Voigt function, the grain size of TiO_2_ is 71.2 nm in BC@TiO_2_-300°C, which is much finer than 87.5 nm in TiO_2_-300°C.

**Figure 3:**
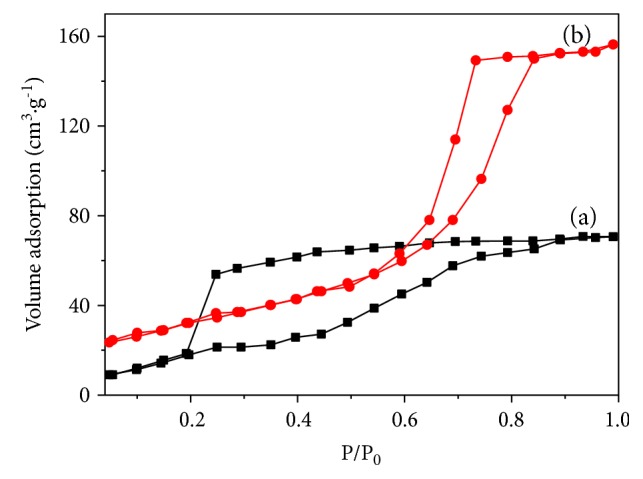
N_2_ adsorption-desorption isotherms at 77 K on (a) BC-300°C and (b) BC@TiO_2_-300°C.

**Figure 4:**
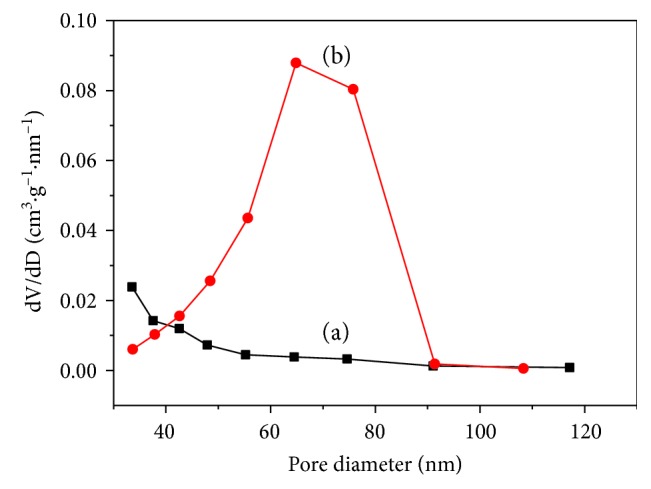
The corresponding pore size distribution of (a) BC-300°C and BC@TiO_2_-300°C determined from the desorption branch of the N_2_ adsorption-desorption isotherms as shown in Figure 3.

Figure 3 shows N_2_ adsorption-desorption isotherms at 77 K on BC-300°C and BC@TiO_2_-300°C. It could be observed obviously that both BC-300°C and BC@TiO_2_-300°C have a hysteresis loop in Figure 3, indicating that all samples have a pore structure. The hysteresis loop of BC@TiO_2_-300°C is at a higher relative pressure than that of BC-300°C, and the decline in the desorption of BC@TiO_2_-300°C is much sharper than that of BC-300°C. It should be an indication of mesoporous or macropore in BC@TiO_2_-300°C, which would be confirmed in Figure 4. Figure 4 shows the corresponding pore size distribution curve determined from the desorption branch of the N_2_ adsorption-desorption isotherms in Figure 3. BC@TiO_2_-300°C exhibits a large average pore diameter of 60 nm and a broad distribution (FWHM = 20 nm). By contrast, BC-300°C shows a pore diameter of less than 40 nm. Correspondingly, the specific surface area of BC@TiO_2_-300°C was calculated to be 161.5m^2^/g by the BET method, larger than 35.0 m^2^/g of BC-300°C. It could be concluded that the pore size of BC is expanded by loading TiO_2_, for the reaming effect of TiO_2_ nanoparticles. Additionally, it is predicted that nanosized TiO_2_ could enter a mean pore diameter of 60 nm in BC@TiO_2_-300°C partly.

**Figure 5:**
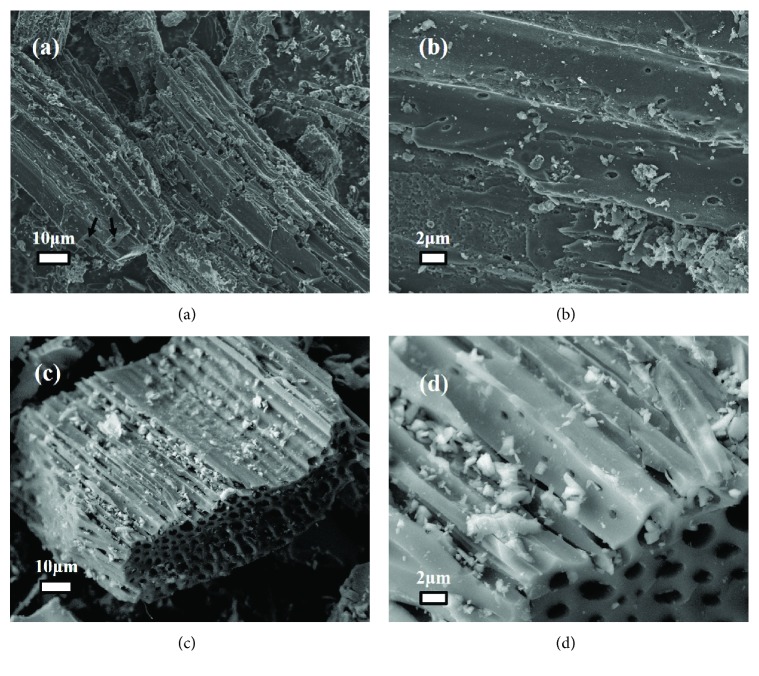
SEM images of (a, b) BC-300°C and (c, d) BC@TiO_2_-300°C.

Figure 5 shows the SEM images of BC-300°C and BC@TiO_2_-300°C. Figures 5(a) and 5(c) reveal that both BC-300°C and BC@TiO_2_-300°C have a rod shape of groove. BC@TiO_2_-300°C presents a well-developed hollow rod with dozens of nanometers and TiO_2_ nanoparticles filled in the hollow structure partly without obvious aggregation as shown in Figures 5(c) and 5(d), In contrast, BC-300°C mostly shows a micropore rod structure as indicated by arrows in Figure 5(a), and several micropores could be observed randomly on the rod wall in Figure 5(b). It is evidenced that pore enlargement of BC has been induced by TiO_2_ loading, consistent with the result of N_2_ adsorption-desorption isotherms analysis. Thus, BC@TiO_2_-300°C should perform better adsorption properties of contaminants than BC-300°C.

**Figure 6:**
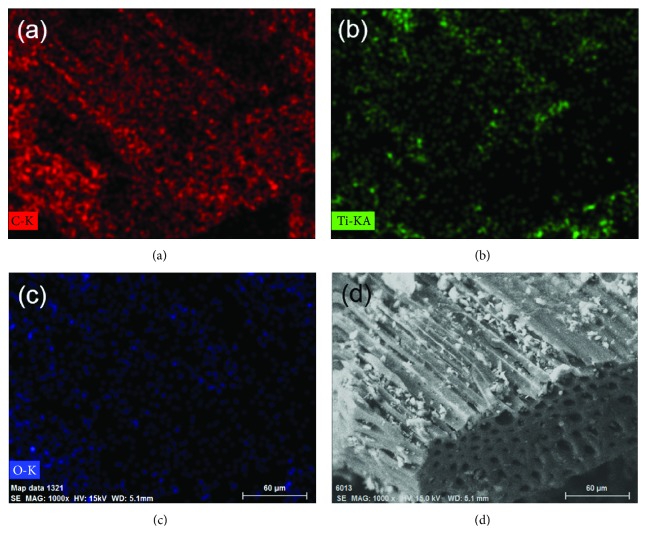
(a–c) Element mapping images and (d) the corresponding SEM image of BC@TiO_2_-300°C.

Figures 6(a)–6(c) show the element mapping images, and Figure 6(d) shows the corresponding SEM images of BC@TiO_2_-300°C. The existence and uniform distribution of Ti and O in BC@TiO_2_-300°C composites are disclosed. Combined with EDS spectrum analysis in Table 1, the loading mass of TiO_2_ could be calculated about 21%. In addition, it should be noted that the stoichiometric ratio of O : Ti reaches 5 : 1, much bigger than 2 : 1 in TiO_2_. It is considered that the O of the organic functional group of BC@TiO_2_-300°C would contribute to its extra content. As discussed above, the organic functional group of BC@TiO_2_ composite would benefit from Congo red removal.

**Figure 7:**
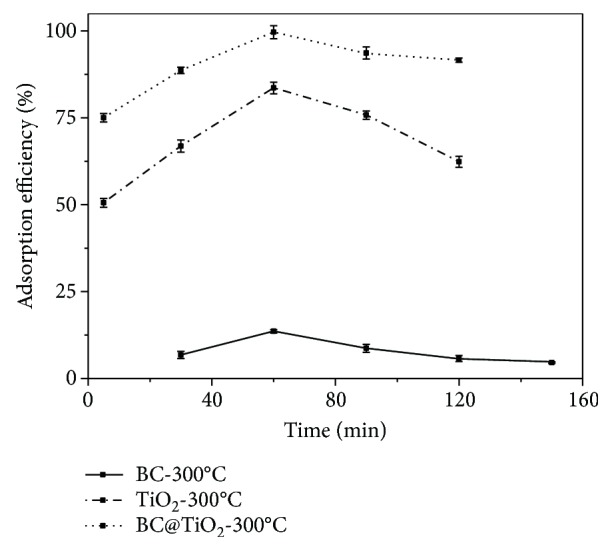
Comparison of the adsorption efficiency for BC-300°C, TiO_2_-300°C, and BC@TiO_2_-300°C.

**Figure 8:**
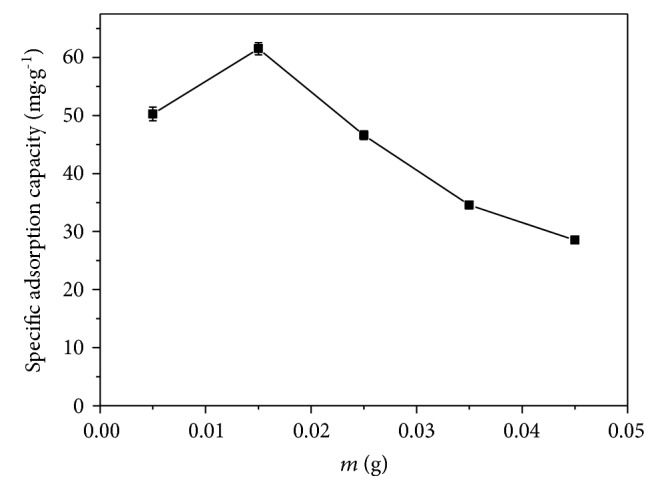
The specific adsorption capacity of BC@TiO_2_-300°C.

Figure 7 compares the relative adsorption capacity for BC-300°C, TiO_2_-300°C, and BC@TiO_2_-300°C. The trend of adsorption performance for all samples looks like the same, rising and reaching saturation in the first 60 min following by declining. The saturated adsorption efficiency for BC@TiO_2_-300°C is enhanced significantly, much more effective than the calculated efficiency of 28.27% by combining with the absorption capacity of 21% TiO_2_ (83.59%) and 79% BC (13.56%). It should be attributed to the synergy effects of physical adsorption in pore-enlarging BC and the weak interaction between the organic functional group of BC@TiO_2_ and the organic molecule of Congo red.  Figure 8 presents the specific adsorption capacity versus the mass of BC@TiO_2_-300°C to remove Congo red. It rises the maximum value of 61.67 mg·g^−1^ when the mass is 0.015 g and then falls down sharply.

**Figure 9:**
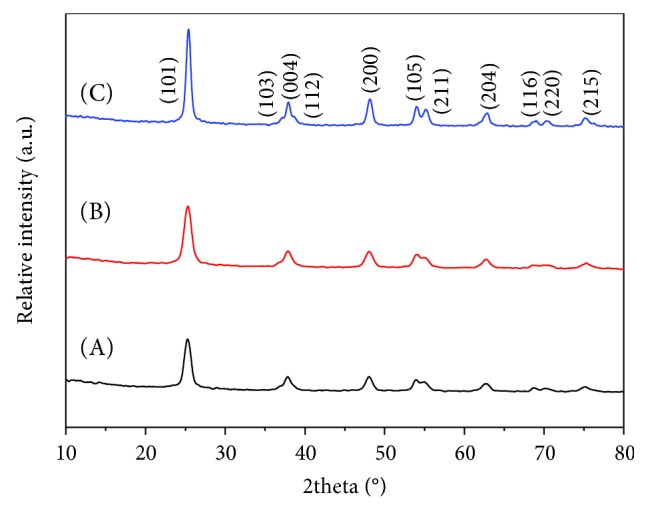
XRD patterns of A BC@TiO_2_-300°C, B BC@TiO_2_-400°C, and C BC@TiO_2_-500°C.

To investigate the synergistic effect variation along with heating temperature, BC@TiO_2_ composites have been prepared at 300°C, 400°C, and 500°C.  Figure 9 shows the XRD patterns of BC@TiO_2_-300°C, BC@TiO_2_-400°C, and BC@TiO_2_-500°C. Consistent with Figure 2, all diffraction peaks belong to anatase TiO_2_ (number 01-084-1286), while the broad diffraction peak of C could not be observed because of an overlapping high crystalline TiO_2_ peak of (101). Along with the temperature increase, the peak intensity increases, and the FWHM narrows, indicating the grain size of TiO_2_ gets larger. Calculated by using a Voigt function, the grain size of BC@TiO_2_-300°C, BC@TiO_2_-400°C, and BC@TiO_2_-500°C is 71.2, 74.3, and 84.3 nm, respectively.

**Figure 10:**
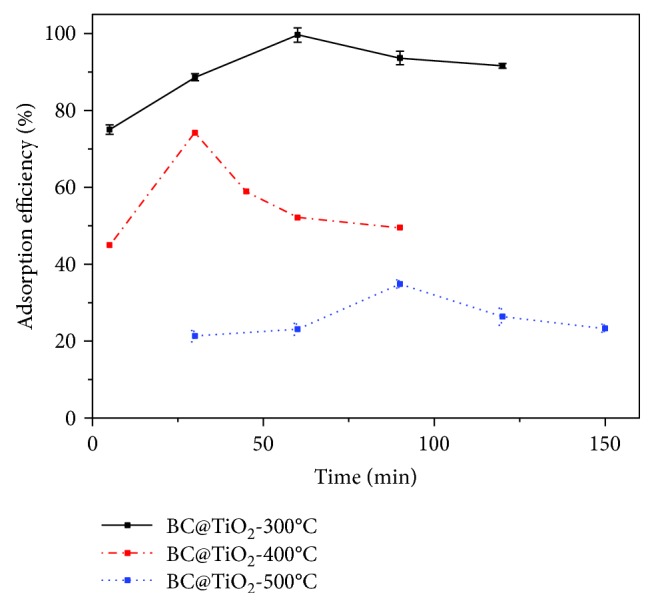
The adsorption efficiency of BC@TiO_2_-300°C, BC@TiO_2_-400°C, and BC@TiO_2_-500°C.

Figure 10 presents the adsorption efficiency of BC@TiO_2_-300°C, BC@TiO_2_-400°C, and BC@TiO_2_-500°C. Different from the results in Figure 7, the adsorption saturation of BC, TiO_2_, and BC@TiO_2_ reaches at the same time of 60 min. It would be observed that BC@TiO_2_-300°C, BC@TiO_2_-400°C, and BC@TiO_2_-500°C reach the adsorption saturation at 60, 30, and 90 min, respectively. In the analysis with the results in Figure 1, it would be considered that the grain size of TiO_2_ has an impact on the saturated time. Additionally, the saturated value of BC@TiO_2_-300°C, BC@TiO_2_-400°C, and BC@TiO_2_-500°C is larger than the same content of BC and TiO_2_. It indicates the existence of the synergistic adsorption effect of BC and TiO_2_ in all BC@TiO_2_-300°C, BC@TiO_2_-400°C, and BC@TiO_2_-500°C. Among them, BC@TiO_2_-300°C shows the best adsorption performance. It would be concluded that the grain size of TiO_2_ is smaller, the adsorption efficiency is higher, but adsorption kinetics is not better. The influence mechanism of adsorption kinetics for BC@TiO_2_ would be further studied.

## 4. Conclusion

In this work, an effective absorbent of BC@TiO_2_ composite has been successfully explored to apply for Congo red removal from aqueous solution. The well-developed hollow pore size of Triarrhena BC is enlarged at a great extent by loading with TiO_2_. Due to the synergistic adsorption effect of BC and TiO_2,_ BC@TiO_2_ shows better adsorption capacity. It could be concluded that it is significant to adjust accurately the pore structure of Triarrhena BC, the grain size of TiO_2_, and the weakly interaction of the functional group between TiO_2_ and BC in order to obtain more excellent adsorption capacity of BC@TiO_2_ composites.

## Figures and Tables

**Table 1:** Element content of BC@TiO_2_-300°C measured by EDS spectrum.

	wt.%	at.%
C	64.44	75.95
O	22.91	20.32
Ti	12.60	3.72

## Data Availability

The data used to support the findings of this study are available from the corresponding author upon request.
